# Incidence of Recurrent Otitis Media With Effusion (OME) Following Tympanostomy in Pediatric Patients: A Systematic Review and Cumulative Meta‐Analysis

**DOI:** 10.1155/ijpe/8886046

**Published:** 2026-06-24

**Authors:** Mohammad Hosein Taziki Balajelini, Mohammad Reza Mofatteh, Masoud Mohammadi, Zahra Taziki Balajelini, Abdolhalim Rajabi

**Affiliations:** ^1^ Department of Surgery, School of Medicine, Golestan University of Medical Sciences, Gorgan, Iran, goums.ac.ir; ^2^ Department of Otorhinolaryngology, School of Medicine, Birjand University of Medical Sciences, Birjand, Iran, bums.ac.ir; ^3^ Golestan Research Center of Gastroenterology and Hepatology, School of Allied Medical Sciences, Golestan University of Medical Sciences, Gorgan, Iran, goums.ac.ir; ^4^ Health Management and Social Development Research Center, Golestan University of Medical Sciences, Gorgan, Iran, goums.ac.ir; ^5^ Department of Biostatistics and Epidemiology, Faculty of Health, Golestan University of Medical Sciences, Gorgan, Iran, goums.ac.ir

**Keywords:** meta-analysis, otitis media with effusion (OME), pediatrics, recurrence, tympanostomy tubes

## Abstract

**Background:**

Otitis media with effusion (OME) is a common pediatric condition, often managed with tympanostomy tube insertion. However, recurrence after tube extrusion remains a significant concern, with global incidence rates varying across studies.

**Objective:**

This systematic review and cumulative meta‐analysis is aimed at quantifying the global incidence of recurrent OME post‐tympanostomy.

**Methods:**

A systematic review and cumulative meta‐analysis was conducted in accordance with the recommendations of the Preferred Reporting Items for Systematic Reviews and Meta‐Analyses (PRISMA). A comprehensive search of electronic databases (PubMed, Web of Science, Embase, Cochrane Library, ProQuest, Wiley, Ovid, and Scopus) was performed in August 2024. The quality of the studies was assessed using the Joanna Briggs Institute (JBI) critical assessment tool. Pooled incidence and 95% confidence intervals were calculated using a random‐effects model. Cumulative meta‐analyses were conducted to track global incidence trends over time. Statistical analysis was conducted using Stata Version 16 software.

**Results:**

From 1117 retrieved articles, 21 met the inclusion criteria. The global pooled incidence of recurrent OME post‐tympanostomy was 30% (95% CI: 23%–37%). Subgroup analysis showed 31% recurrence with tubes alone (95% CI: 24%–38%) versus 20% with adjunctive antibiotics (95% CI: 14%–26%). The highest recurrence (45%, 95% CI: 34%–55%) occurred in cases combining tympanostomy with cleft palate surgery. Cumulative meta‐analysis indicated a declining trend in OME recurrence between 2005 and 2019, with the incidence rate decreasing from 39% to 24% over this period.

**Conclusions:**

Approximately one‐third of children undergoing ventilation tube (VT) insertion surgery experience recurrence of OME. Although some studies suggest that adding antibiotics to VT insertion therapy may reduce recurrence in children at risk for chronic infection, this evidence is not consistent and should be interpreted with caution. In addition, patients with cleft palate have a higher likelihood of OME recurrence and require long‐term follow‐up. These findings may assist physicians in refining treatment decisions based on individual patient characteristics and recurrence risk, though further high‐quality clinical trials are needed to confirm these observations.

## 1. Introduction

Otitis media with effusion (OME) or serous otitis media (SOM) is defined as the accumulation of fluid in the middle ear cavity that occurs chronically without acute symptoms [1, 2]. This disease is one of the most common ear disorders in children, with about 80% of children suffering from it at least once before entering school [3, 4]. However, it is difficult to determine the exact incidence of this disease because many patients are asymptomatic. The prevalence of OME varies depending on the age of the patient, the season of the year, and the method of assessment. Studies have shown that the prevalence of this disease in children is between 15% and 20%, and about 90% of preschool‐aged children have experienced it at least once [1]. About 65% of OME cases in children aged 2–7 years recover within a month, but 30%–40% of children may experience repeated relapses, and in 5%–10% of cases, the disease may persist for up to a year [1, 5].

Late diagnosis of OME can lead to complications such as tympanic membrane retraction, tympanosclerosis, adhesive otitis media, and hearing and speech disorders [5, 6]. Several risk factors for developing this disease have been identified, including a family history of otitis media, tobacco smoke exposure, low socioeconomic status, adenoid hypertrophy, attendance at a day care center, immune system disorders, cleft palate, cystic fibrosis, allergies, vitamin D deficiency, and gastroesophageal reflux [7–9].

OME is primarily caused by viral infections or allergies rather than bacterial infections. As a result, antibiotics are not recommended for treatment. Additionally, the use of corticosteroids for allergy management has not been shown to significantly improve OME outcomes. For these reasons, neither antibiotics nor corticosteroids are advised for treating OME. The recommended first‐line approach is watchful waiting for 3 months. If OME persists beyond this period, a referral to a specialist may be considered to evaluate potential surgical treatment options [10]. In other words, in cases where conservative treatments are not effective, surgical procedures such as myringotomy and placement of a ventilation tube (VT) are recommended [11]. Patients who do not improve after 3 months despite conservative treatments are candidates for VT placement [12, 13]. VTs are extruded spontaneously after a while, and the eardrum heals, although the time it takes for the tubes to be extruded varies [14].

Despite surgery and medical treatments, there is a possibility of OME recurrence, and the recurrence rate has been reported to be between 19% and 40% [15–18]. Factors affecting disease recurrence include patient age, gender, exposure to cigarette smoke, allergies, breastfeeding history, and maternal education level [15, 19, 20]. Also, cleft palate is known to be an underlying factor that significantly increases the likelihood of recurrence [21, 22]. The type of VT used and the patient′s age at the time of surgery are other factors affecting disease recurrence [23].

In different studies, the incidence of recurrent OME in children has been reported to vary considerably, and several factors associated with it have been identified. The clinical significance of recurrent OME in hearing loss, the impact on speech development, and behavior in children highlight the need for early identification of high‐risk groups and associated factors. However, a comprehensive systematic review and meta‐analysis on the incidence of recurrent OME after surgery has not been conducted worldwide. Therefore, considering the need for strong, synthesized evidence to support clinical decision‐making and to understand the causes and contributing factors of recurrence in patients with OME who have undergone surgery, this study systematically reviews and performs a meta‐analysis on the global incidence of recurrent OME following tympanostomy in pediatric patients.

## 2. Methods

### 2.1. Literature Search

A systematic review of the existing scientific literature was undertaken to identify original studies investigating the incidence of SOM. This review adhered to the guidelines established by the 2020 Preferred Reporting Items for Systematic Reviews and Meta‐Analyses (PRISMA) statement (Supporting Information 1: Appendix S1). The protocol for this systematic review and meta‐analysis was not registered in PROSPERO or any other platform. The systematic literature search was performed up to August 2024 and encompassed eight English‐language digital databases, including PubMed, Web of Science, Embase, Cochrane Library, ProQuest, Wiley, Ovid, and Scopus.

The search terms utilized in this review included “Incidence,” “Risk,” “Prevalence,” “Person‐Time Rate,” “Serous Otitis Media,” “Secretory Otitis Media,” “Otitis Media with Effusion,” “Recurrence,” and “Relapse.” These terms were employed to formulate search strings, which were subsequently combined in various configurations using Boolean operators such as “AND” and “OR.” Special attention was given to studies addressing the topic of recurrence, and a manual search of relevant reference lists was conducted. The full texts of the identified publications were meticulously screened for primary data. The detailed search strategies for each database are provided in Supporting Information 2: Appendix S2.

To manage references and remove duplicates, all retrieved records were imported into EndNote software. Data extraction was independently conducted by two trained reviewers (M.M. and Z.T.B.), who performed a comprehensive assessment of each included study to confirm data validity and participant eligibility. The extracted dataset encompassed study‐level and participant‐level variables: publication year, first author, geographical and clinical context, study design, presence of concomitant surgical procedures, type of intervention, participant age, follow‐up duration, total sample size, and incidence of SOM. Any disagreements that emerged during the interpretation of the data were resolved through consultative discussions between the two researchers. Any inconsistencies between the two reviewers were addressed through structured discussions until consensus was reached. In this study, recurrent OME was defined as the reappearance of clinical manifestations—such as fluid or air bubbles behind the tympanic membrane or evidence of effusion—following the spontaneous extrusion of tympanostomy tubes.

### 2.2. Inclusion and Exclusion Criteria

Inclusion criteria are as follows: (1) the studies must be original cohort studies, randomized controlled trials (RCTs), or cross‐sectional studies; (2) the studies must clearly define the therapeutic intervention; (3) all studies involved initial VT placement; and (4) the studies must exclusively focus on SOM.

Exclusion criteria are as follows: (1) experimental animal studies; (2) literature that is not relevant to the study topic; (3) studies that did not report the number of incidents of SOM; and (4) publications that include reviews, conference papers, commentaries, or case reports.

### 2.3. Risk of Bias

The quality of the included studies was assessed by two independent investigators (Z.T.B. and A.R.) utilizing the Joanna Briggs Institute (JBI) tool, which comprises nine items aimed at evaluating the methodological quality of a study and assessing the extent to which potential bias was addressed in its design, conduct, and analysis [24]. The JBI score ranges from 0 to 9, with higher scores indicating a lower risk of bias. Any discrepancies in the assessment were resolved through discussion or by consulting the third investigator (M.H.T.B.).

### 2.4. Statistical Analysis

All statistical analyses were performed using Stata software (Version 16.0; StataCorp LP, College Station, Texas, United States). Given the considerable heterogeneity observed across the studies, a random‐effects model was utilized to aggregate the estimated incidences from the included studies. Heterogeneity was quantified using the I^2^ statistic, and visual inspection of the forest plots assisted in assessing its presence. The results are displayed in forest plots accompanied by 95% confidence intervals (CIs). A significance threshold of *p* < 0.05 was adopted for all analyses. Additionally, stratified analyses were undertaken based on study type, treatment modality, the presence of concomitant surgical procedures, and geographical continent. To evaluate the impact of mean age at study enrollment, mean follow‐up duration for recurrence, and study quality scores on the incidence of SOM, a meta‐regression analysis was performed. Furthermore, the presence and implications of publication bias were examined through visual inspection of funnel plots alongside Begg′s and Egger′s tests. A trim‐and‐fill analysis was also conducted to evaluate the robustness of the overall incidence estimates in light of any identified publication bias. Cumulative meta‐analyses were utilized to assess the differential accumulation of evidence regarding the incidence of specified outcomes globally. These cumulative meta‐analyses provide pooled estimates and 95% CIs, recalculating the overall incidence and CIs as new studies are incorporated, thereby illustrating the temporal evolution of incidence rates.

## 3. Results

**Figure 1:**
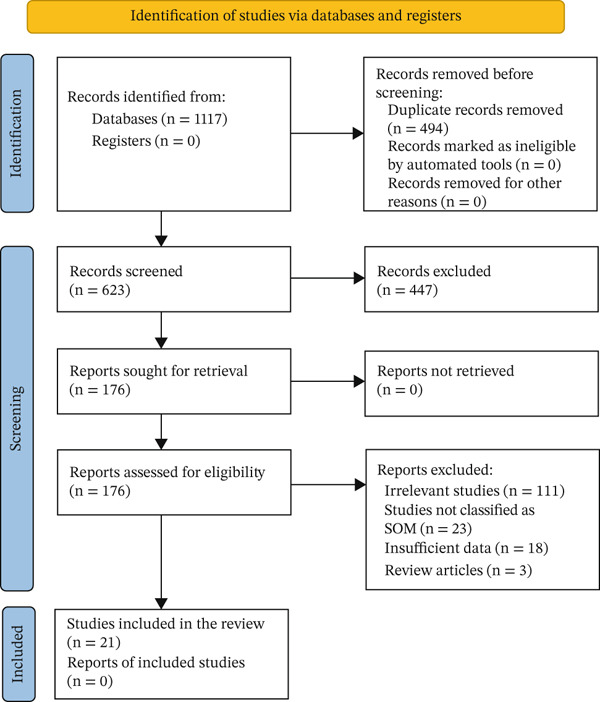
PRISMA flow diagram of studies included in the meta‐analysis.

As represented in Figure 1, an initial search employing a keyword‐based strategy returned 1117 potentially relevant articles. Following the removal of 494 duplicates, 623 articles underwent a preliminary screening based on their titles and abstracts. Of these, 447 were excluded due to several factors: incongruence with the study′s objective, utilization of animal models, or classification as systematic or narrative reviews. A subsequent in‐depth full‐text review of the remaining 176 articles resulted in the exclusion of 155 articles that did not meet our inclusion criteria, with the following reasons for exclusion: (1) irrelevant study content (*n* = 111); (2) non‐SOM status (*n* = 23); (3) insufficient data provided for incidence calculation (*n* = 18); and (4) classification as a review article (*n* = 3). After rigorous screening, 21 articles were ultimately included in our analysis. Any contentious points arising during this process were discussed and resolved by the research team members.

This systematic review included 21 studies published between 2000 and 2024. These studies were conducted across multiple continents, including Asia (Iran, Japan, South Korea, Thailand, Saudi Arabia, and China), Europe (Denmark, France, Greece, Italy, Netherlands, Serbia, Turkey, and Bulgaria), North America (United States), and Africa (Egypt). Regarding methodology, 18 studies employed a cohort design, whereas three utilized an interventional design (clinical trials). The mean age of participants across the studies ranged from 1.14 to 10 years. The follow‐up period for patients after treatment varied from 3 to 104 months. Concerning therapeutic interventions, 19 studies focused solely on the treatment of OME using VT insertion. Only two studies combined VT with topical antibiotics for OME treatment. Furthermore, in 11 studies, VT surgery was performed alone. In seven other studies, adenotonsillectomy was performed concurrently with VT. In three studies, cleft palate surgery was performed alongside VT. Additional details of the studies included for meta‐analysis are presented in Table 1.

**Table 1:** Characteristics of included studies.

Author (publication year)	Country	Continent	Study design	Accompanying surgery	Quality score	Type of intervention	Population	Age	Follow‐up time
Saad et al. (2021) [25]	Egypt	Africa	Cohort study	Tonsillectomy	7	VT	Patients with OME received a tympanostomy tube either bilaterally or unilaterally	7.13 ± 1.30	24
Yaman et al. (2010) [26]	Turkey	Europe	Cohort study	NR	6	VT	Children with chronic OME who underwent tympanostomy tube insertion	7.59 ± 3.14	Median 23.21 ± 14.58 months
De Felice et al. (2008) [27]	Italy	Europe	Cohort study	NR	6	VT	OME cohort, very low birth weight (VLBW) infants	9.5 ± 2.8	3 years
Engel et al. (2005) [28]	Netherlands	Europe	Cohort study	NR	7	VT	Children aged between 2 and 7 years with a first clinical episode of bilateral OME	5.3, R: 2.1–7.5	3 and 27 months
Xenellis et al. (2005) [29]	Greece	Europe	Cohort study	Adenoidectomy	7	Antibiotics + VT	Children with unilateral or bilateral OME	6–12 years	16 months
Ovesen et al. (2000) [30]	Denmark	Europe	Clinical trial	NR	5	Antibiotics + VT	Undergoing VT insertion bilaterally, 1–7 years	Mean: 38 months (1–7 years)	11–39 months
Popova et al. (2010) [31]	Bulgaria	Europe	Clinical trial	Adenoidectomy	6	VT	Children with documented history of bilateral middle ear effusion for at least 3 months and conductive hearing loss greater than 20 dB	3–7 years	12 months
Hao et al. (2019) [18]	China	Asia	Clinical trial	Adenoidectomy	7	VT	Children treated by tube insertion and adenoidectomy and tympanostomy tube insertion	5.03 ± 0.95	1.5–3.5 years
Klopp‐Dutote et al. (2018) [16]	France	Europe	Cohort study	NR	6	VT	Children having undergone bilateral placement of a tympanostomy tube	Under 12 years	4 years
Baljošević et al. (2017) [32]	Serbia	Europe	Cohort study	Adenoidectomy	7	VT	Children with OME treated by the same senior surgeon	0–10 years	12 months
Rieu‐Chevreau et al. (2019) [33]	France	Europe	Cohort study	Cleft palate surgery	7	VT	Children treated for cleft palate in a French university medical center	7–13 years	104 months
Iemura‐Kashiwagi et al. (2022) [34]	Japan	Asia	Cohort study	Cleft palate surgery	6	VT	Pediatric patients who underwent palatoplasty for cleft palate	13.7 months	7–48 months
Luo et al. (2014) [35]	China	Asia	Cohort study	NR	6	VT	Children diagnosed with OME and adenoid hypertrophy	4.7 ± 1.2	2 years
Suwansa‐Ard et al. (2023) [36]	Thailand	Asia	Cohort study	Cleft palate surgery	5	VT	All nonsyndromic cleft palate patients, including the OME patients	5.8 ± 2.8	1 week–1 month–every 3 months
Shareef et al. (2024) [37]	United States	America	Cohort study	Adenoidectomy	6	VT	Children undergoing (initial) tympanostomy tube placement at Dayton Children′s Hospital	Med: 18 (range: 5–48) months	1–8 years
Ha et al. (2023) [38]	South Korea	Asia	Cohort study	NR	4	VT	Pediatric patients with cleft palate who underwent both palatoplasty and tympanostomy tube surgery	1.18 years (22.1 ± 25.7)	6 months
Jia et al. (2023) [39]	China	Asia	Cohort study	Adenoidectomy	5	VT	Pediatric patients who underwent BET + VT insertion, as well as patients who underwent VT insertion alone	7.69 ± 2.27	12 months
Takai et al. (2023) [13]	Japan	Asia	Cohort study	NR	7	VT	Children with persistent OME who underwent the Koken B‐type VT insertion	3.4 (0.4–15) years	32
Alaraifi et al. (2020) [40]	Saudi Arabia	Asia	Cohort study	NR	7	VT	Children who underwent unilateral or bilateral VT insertion for treatment of OME	6.15 (±3.64)	For 12–18 months
Straetemans et al. (2005) [41]	Netherlands	Europe	Cohort study	NR	6	VT	Children received the same type of tympanostomy tube bilaterally	Med: 6.3 (range: 3.4–9.1)	6
Taziki et al. (2024) [42]	Iran	Asia	Cohort study	NR	7	VT	Children underwent myringotomy and tympanostomy tube insertion surgery	6.24 ± 2.66	11.36 ± 16.11

### 3.1. Risk of Bias

Overall, the mean study quality score was 6.19 ± 0.87, with a minimum score of 4 and a maximum of 7. Detailed study quality assessment results are presented in Table 1.

### 3.2. Meta‐Analysis

**Figure 2:**
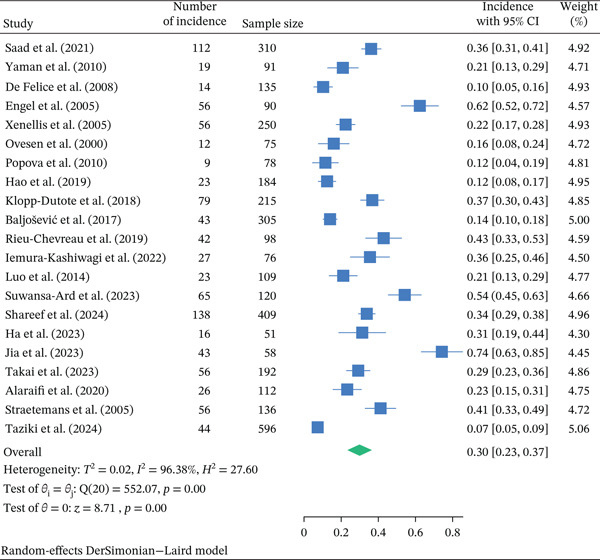
Overall incidence of OME recurrence.

A total of 21 studies, encompassing 3690 patients with OME who underwent therapeutic intervention, were included in this meta‐analysis to assess the incidence of OME recurrence. The overall meta‐analysis revealed an approximate 30% recurrence rate following intervention (incidence = 0.3, 95% CI: 0.23–0.37). These findings also indicate substantial heterogeneity among the study results, as evidenced by an I^2^ value of 96.38% (Figure 2).

### 3.3. Subgroup Meta‐Analysis

**Figure 3:**
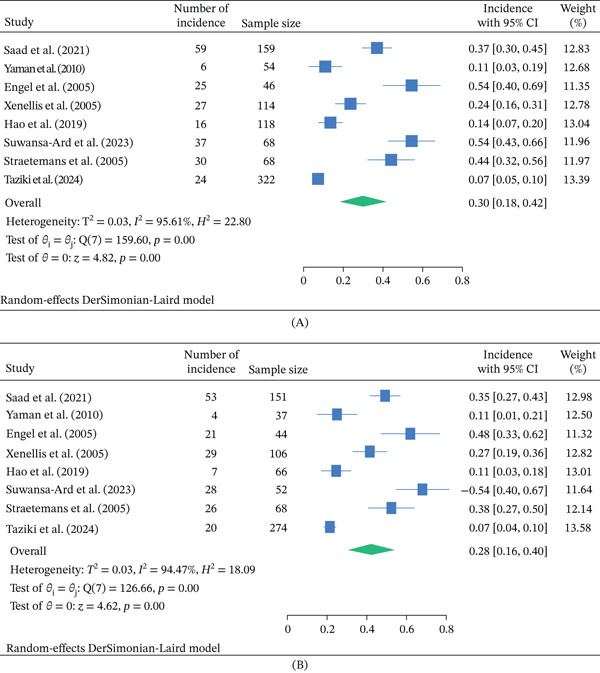
Overall incidence of OME recurrence by sex. (A) Boys. (B) Girls.

Subgroup analysis based on gender revealed that a total of eight studies reported the incidence of OME recurrence in boys, and eight studies reported it in girls. The remaining studies did not provide gender‐specific incidence rates. Meta‐analysis results for these subgroups demonstrated an OME recurrence rate of 30% in boys (incidence = 0.3, 95% CI: 0.18–0.42) and 28% in girls (incidence = 0.28, 95% CI: 0.16–0.40) (Figure 3).

**Figure 4:**
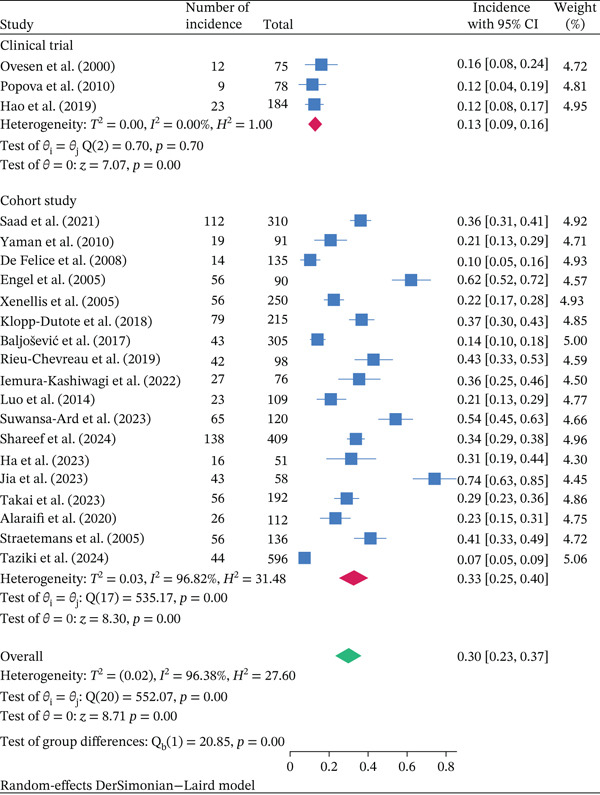
Incidence of OME recurrence by study design.

Subgroup analysis based on study design indicated that a total of 18 studies were conducted using a cohort design (mean follow‐up: 26.05 ± 23.98), and three studies employed an interventional design (clinical trials, mean follow‐up: 22.33 ± 9.29). Meta‐analysis results for these subgroups revealed an OME recurrence rate of 13% in interventional studies (incidence = 0.13, 95% CI: 0.09–0.16) and 33% in cohort studies (incidence = 0.33, 95% CI: 0.25–0.40) (Figure 4).

**Figure 5:**
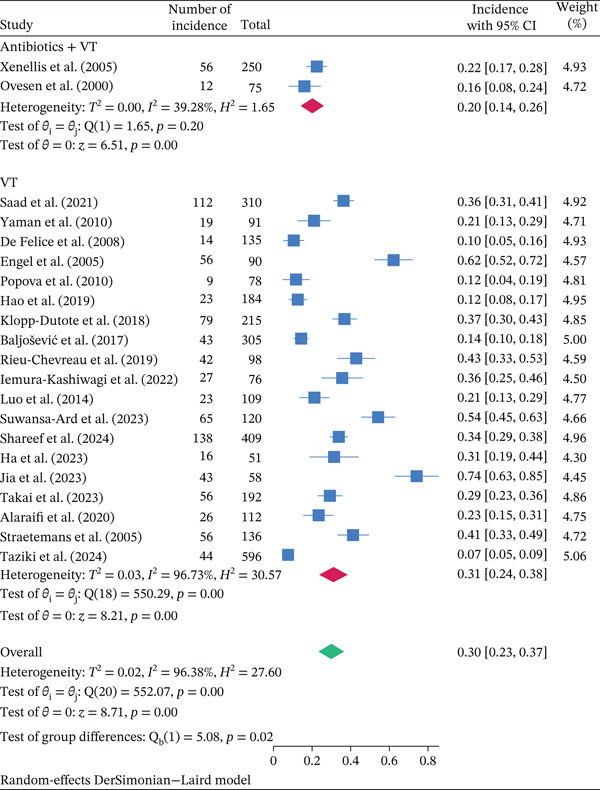
Incidence of OME recurrence by treatment intervention.

Subgroup analysis based on treatment intervention revealed that 19 studies utilized VT insertion for the treatment of OME, and two studies combined VT with topical antibiotics. Meta‐analysis results for these subgroups showed an OME recurrence rate of 31% in studies using VT alone (incidence = 0.31, 95% CI: 0.24–0.38) and 20% in studies combining VT with topical antibiotics (incidence = 0.20, 95% CI: 0.14–0.26) (Figure 5).

**Figure 6:**
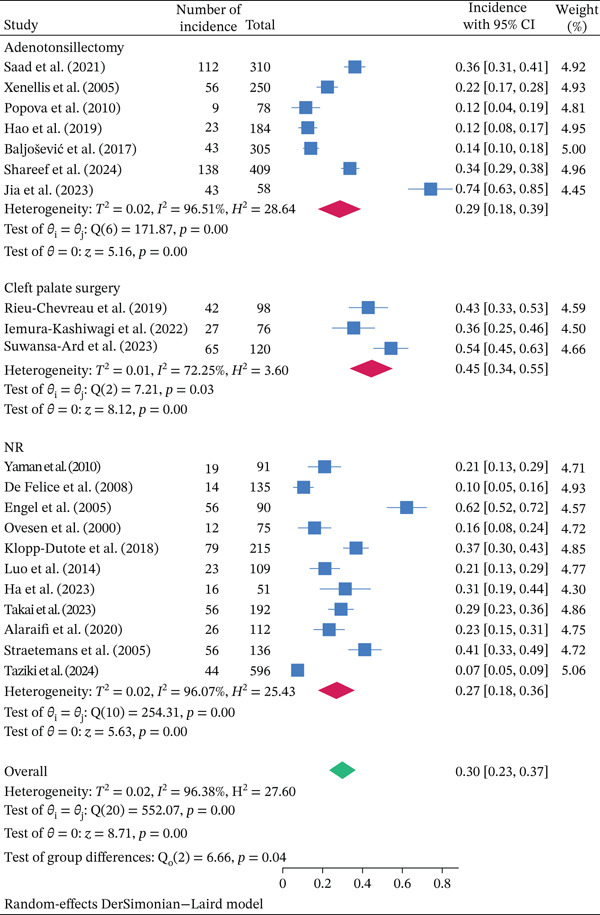
Incidence of OME recurrence by concomitant surgical procedure.

Subgroup analysis based on the type of surgical procedure revealed that seven studies performed adenotonsillectomy in conjunction with VT insertion, three studies performed cleft palate surgery alongside VT, and 11 studies performed VT insertion alone. Meta‐analysis results for studies that performed adenotonsillectomy with VT showed an OME recurrence rate of 29% (incidence = 0.29, 95% CI: 0.18–0.39). Among studies that performed cleft palate surgery with VT, the OME recurrence rate was 45% (incidence = 0.45, 95% CI: 0.34–0.55). In contrast, studies that solely utilized VT insertion reported an OME recurrence rate of 27% (incidence = 0.27, 95% CI: 0.18–0.36) (Figure 6).

**Figure 7:**
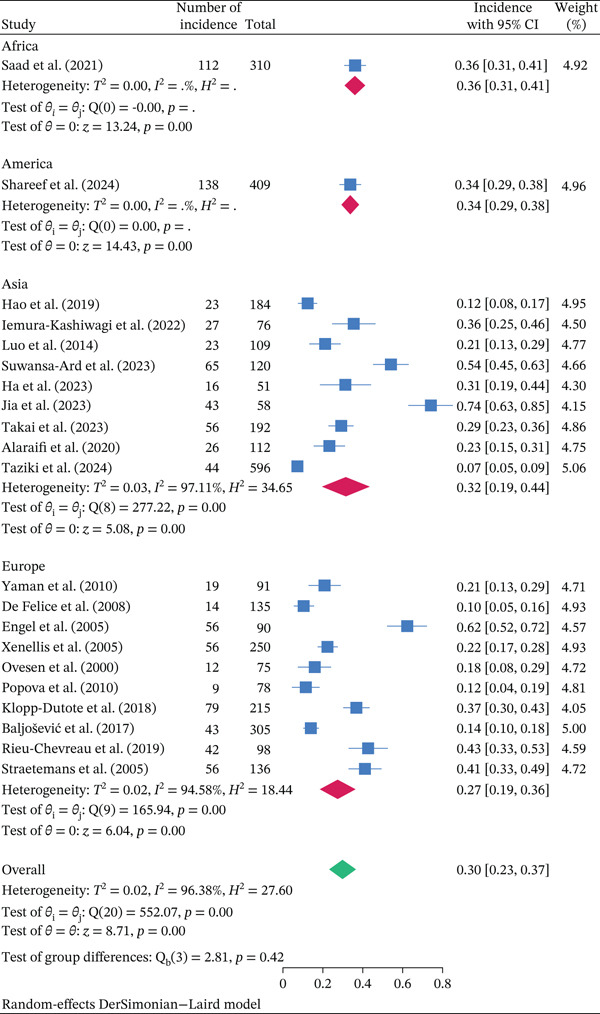
Incidence of OME recurrence by continent.

Subgroup analysis based on the study continent revealed that nine studies were conducted in Asian countries, 10 studies in European countries, and one study each in North America and Africa. Meta‐analysis results showed an OME recurrence rate of 32% in Asia (incidence = 0.32, 95% CI: 0.19–0.44) and 27% in Europe (incidence = 0.27, 95% CI: 0.19–0.36) (Figure 7).

### 3.4. Cumulative Meta‐Analysis

**Figure 8:**
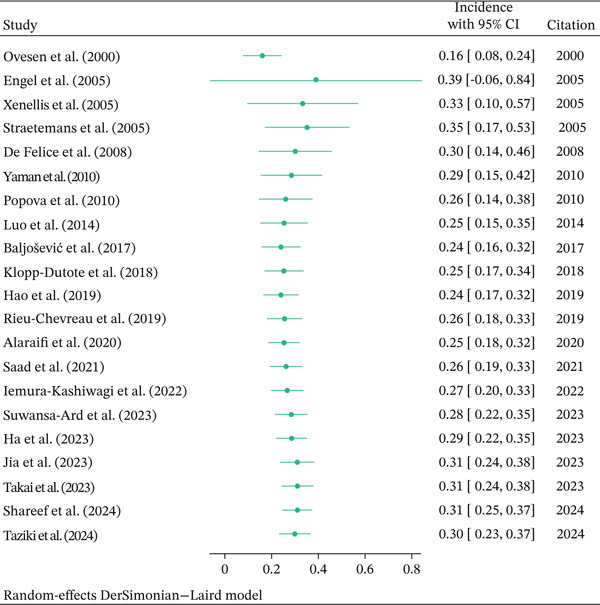
Cumulative meta‐analysis of OME recurrence incidence.

Based on the included studies, cumulative meta‐analysis demonstrated a decreasing trend in OME recurrence between 2005 and 2019, with the recurrence rate declining from 39% to 24%. However, cumulative meta‐analysis of studies from 2019 to 2024 revealed an increasing trend in OME recurrence (Figure 8).

### 3.5. Meta‐Regression Analysis

**Figure 9:**
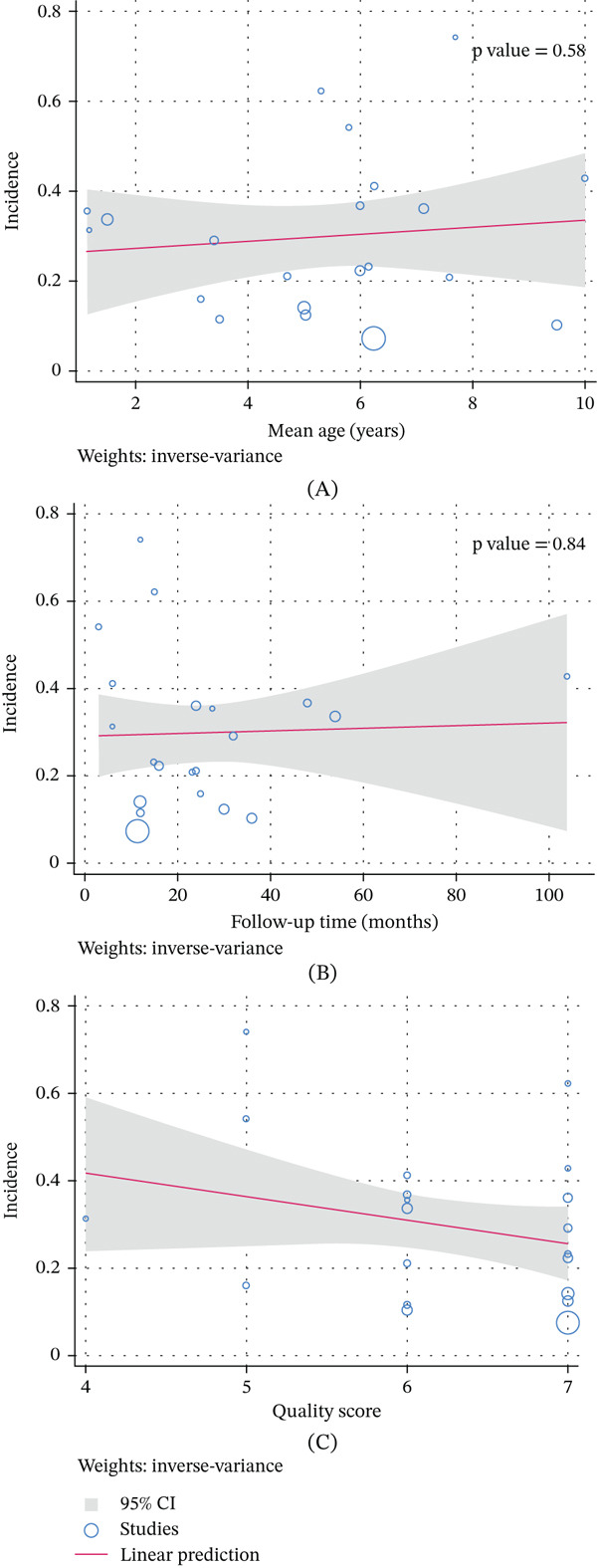
Meta‐regression of OME recurrence based on (A) mean age of patients, (B) follow‐up duration (months), and (C) quality of studies.

Meta‐regression analysis revealed that the variables of mean age in the studies, mean follow‐up time for recurrence, and study quality scores did not have a statistically significant effect on the variations in OME incidence across the studies (Figure 9).

### 3.6. Publication Bias

**Figure 10:**
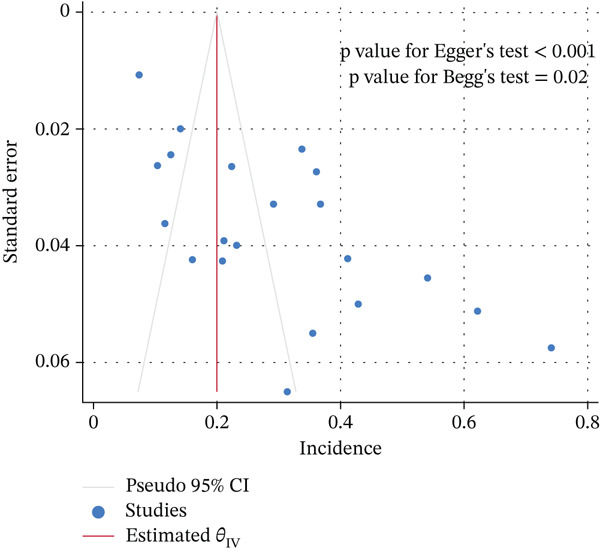
Funnel plot for assessing publication bias in OME recurrence.

Publication bias was assessed using a funnel plot. Visual inspection of the funnel plot suggested the presence of publication bias, which was further supported by the statistical results of Egger′s regression test (*p* < 0.001) and Begg′s test (*p* = 0.02). However, the results remained robust after applying the trim‐and‐fill analysis (Figure 10).

## 4. Discussion

OME recurrence is a common complication following tympanostomy tube removal in children. Some studies indicate that up to 50% of children undergoing surgery for chronic OME experience recurrence, necessitating further interventions [43, 44]. This study was conducted as a systematic review and meta‐analysis of 21 observational and interventional studies, encompassing 3690 individuals who underwent VT insertion. The objective of this research was to investigate the incidence of OME recurrence in these individuals. To the best of our knowledge, this is the first systematic review and meta‐analysis to examine the incidence of OME recurrence among children undergoing VT insertion.

This study demonstrated that the global incidence of OME recurrence among patients who underwent surgery for OME was 30% (95% CI: 23%–37%). This incidence approximates the global mean incidence reported across various studies [41, 45, 46]. However, due to the substantial heterogeneity observed in the reported OME recurrence incidence across the 21 studies included in our meta‐analysis, our pooled estimates should be interpreted with caution. Subgroup analyses were conducted to investigate the incidence of OME recurrence following VT insertion across different variables to identify potential sources of this heterogeneity.

The OME recurrence incidence in the present study demonstrates similar rates in boys and girls, with 30% (95% CI: 18%–42%) and 28% (95% CI: 16%–40%), respectively. This negligible difference in OME recurrence incidence between the two genders aligns well with the results of previous studies. Analyses conducted by researchers such as Yaman et al. [26], Saad et al. [25], Rovers et al. [47], and Taziki et al. [42] have shown no statistically significant difference in OME recurrence incidence between boys and girls. These findings emphasize the need for similar strategies in managing OME recurrence for both genders and can contribute to effective clinical decision‐making.

Analysis of OME recurrence based on study design revealed a recurrence rate of 33% in cohort studies and 13% in clinical trials. It is noteworthy that the meta‐analysis of cohort studies demonstrated substantial heterogeneity among the primary studies. Furthermore, due to the observational nature of these studies, the potential for residual confounding factors exists [48], which may influence the findings. In contrast, the meta‐analysis of clinical trials showed no observed heterogeneity among the primary studies. As confounding factors are controlled at the study onset through randomization in these types of studies [49], the likelihood of their influence is reduced, and the results from these studies are considered more valid. However, it should be noted that the number of clinical trials included in this meta‐analysis was limited. Despite the absence of a statistically significant difference (*p* = 0.79), the longer mean follow‐up duration in cohort studies (26.05 months) compared with intervention studies (22.33 months) may have influenced the observed differences in recurrence rates. Therefore, the interpretation of these results should be approached with caution, and further clinical trials are necessary to confirm these findings. Importantly, the analysis of recurrence rates across study designs was conducted solely for descriptive purposes to illustrate subgroup findings and was not intended as a direct comparative evaluation between cohort studies and clinical trials.

Meta‐analysis results of primary studies based on treatment intervention, which included two studies using VT insertion combined with antibiotics for OME treatment and 19 studies using VT insertion alone, showed that the OME recurrence rate was 31% in studies using VT insertion alone, whereas it was 20% in studies using VT insertion combined with antibiotics. These results demonstrated a statistically significant difference. This significant difference in OME recurrence rates between the treatment groups may be attributed to the different mechanisms of action of the interventions. VTs play a crucial role in OME treatment by improving middle ear ventilation, reducing negative pressure, and preventing fluid accumulation. However, residual inflammation or bacterial infections may lead to recurrence in some patients. The addition of antibiotics to VT treatment likely provides greater protection against OME recurrence by reducing bacterial load and chronic inflammation in the middle ear. Studies have shown that bacterial infections are significant factors in OME persistence and recurrence [50–52]; thus, the combination of these two methods may reduce recurrence rates. These results suggest that in patients at higher risk for recurrence, particularly those with suspected chronic infection, antibiotic administration alongside VT may be a more effective strategy. However, given concerns regarding antibiotic resistance, caution should be exercised in selecting this treatment approach, and it should only be used in eligible patients. Therefore, the decision to use antibiotics in conjunction with VT should be based on a thorough assessment of the patient′s condition, recurrence risk, and other clinical factors. It is noteworthy that the number of studies examining the combined effect of VT and antibiotics is limited, and the results are not conclusive; thus, these results should be interpreted with caution.

Also, the interpretation of recurrence rates in this review requires consideration of factors beyond the tympanostomy tube procedure itself. Specifically, the condition and management of the adenoids and tonsils play a pivotal role in long‐term outcomes. Adenoid hypertrophy is known to impair Eustachian tube function, thereby increasing susceptibility to persistent or recurrent OME. Evidence from previous studies indicates that adenoidectomy, when clinically warranted, substantially reduces the risk of recurrent or persistent middle ear effusion and enhances the durability of tympanostomy tube effectiveness [53–55]. In addition, recurrent adenotonsillar infections and associated lymphoid hypertrophy may perpetuate chronic nasopharyngeal inflammation, further compromising middle ear ventilation [56].

Meta‐analysis results of primary studies based on the presence of concomitant surgical procedures with VT insertion revealed that three studies included patients who underwent cleft palate surgery, seven studies included patients who underwent adenotonsillectomy, and 11 studies did not specify any concomitant surgical procedures. Data analysis showed that the highest OME recurrence rate was observed in studies where patients underwent cleft palate surgery (45%), whereas it was 27% in studies where adenotonsillectomy was performed. This difference was reported to be statistically significant. The variation in OME recurrence rates based on the type of concomitant surgery may be attributed to related anatomical and physiological factors. In patients with cleft palate, inefficient function of the Eustachian tube dilator muscles leads to poor middle ear ventilation and an increased likelihood of fluid accumulation. This structural defect, even after VT insertion, can be a significant factor in increasing OME recurrence rates [33, 57], which aligns with the findings of the present meta‐analysis (45% recurrence incidence). In contrast, patients undergoing adenotonsillectomy likely experience improved middle ear ventilation due to reduced chronic inflammation and upper airway obstruction. This may explain the lower OME recurrence rate (27%) observed in this group. Previous studies have shown that adenotonsillectomy helps improve Eustachian tube function and reduces the likelihood of OME recurrence [15, 58]. These results indicate that in patients with cleft palate, even after VT insertion and subsequent removal, the likelihood of OME recurrence remains high, necessitating long‐term follow‐up and further therapeutic interventions. Conversely, in patients with adenoid and tonsillar hypertrophy, adenotonsillectomy may be a potential strategy to reduce OME recurrence and improve middle ear status. These findings can be useful in clinical decision‐making for selecting appropriate treatment modalities based on patient conditions. However, it is important to note that both subgroups exhibited substantial statistical heterogeneity.

Meta‐analysis results of OME recurrence incidence based on study continent revealed rates of 32% in Asia and 27% in Europe. Statistical analysis did not show a significant difference between these two continents. Regarding Africa and North America, only one study was conducted in each, and due to the limited number of studies, their results cannot be reliably extrapolated. These findings suggest that OME recurrence may be influenced by various factors, including genetic and environmental characteristics, public health status, access to medical services, and treatment practices. The lack of a statistically significant difference in recurrence rates between Asia (32%) and Europe (27%) may indicate that this condition is primarily influenced by shared physiological and pathophysiological factors across different populations, rather than solely by geographical location. However, environmental factors such as air pollution, nutritional status, exposure to infectious agents, and population density may play a significant role in OME incidence and recurrence. Furthermore, the limited number of studies in Africa and North America prevents definitive conclusions regarding the impact of geographical location on this condition. Therefore, to achieve a comprehensive conclusion, more studies with larger sample sizes and broader coverage in various regions of the world are needed to more accurately assess the impact of geographical and environmental factors on OME recurrence.

Cumulative meta‐analysis revealed a decreasing trend in OME recurrence among patients undergoing VT insertion between 2005 and 2019, with a reduction from 39% to 24%. This decrease may be attributed to improvements in treatment modalities, such as the wider use of VTs and antibiotics, and advancements in the management of infectious and allergic diseases. Additionally, increased awareness regarding early diagnosis and appropriate treatment of OME may have contributed to the reduction in recurrence rates during this period. However, the increasing trend in OME recurrence from 2019 to 2024 could be due to several factors. These factors may include the increased prevalence of viral respiratory diseases, such as COVID‐19, which can lead to middle ear complications, or changes in treatment practices and antibiotic resistance. Furthermore, environmental factors such as air pollution, climate change, and genetic factors may play a role. Therefore, changes in the OME recurrence trend may be dependent on a combination of factors that require more detailed and continuous investigation.

Furthermore, the results of the present study indicated that OME recurrence was not associated with factors such as patient age at the time of surgery or follow‐up duration after VT insertion. These findings suggest that current treatments for OME, such as VTs, generally have a similar effect regardless of age and posttreatment follow‐up duration. Therefore, decisions regarding the use of treatment modalities and posttreatment follow‐ups should be based more on individual patient clinical characteristics and clinical evidence related to recurrent infections and middle ear problems, rather than solely on age or follow‐up duration.

To our knowledge, this study represents the first systematic review and meta‐analysis focused on OME recurrence in children undergoing VT insertion. The results obtained from subgroup analyses indicate the robustness of the OME recurrence estimates. However, several limitations in this study should be considered. First, the power of subgroup analyses was limited due to the small number of studies in certain categories (e.g., only two studies on VT plus antibiotics vs. VT alone, three studies in patients with cleft palate, and only one study each from Africa and North America), which restricts the generalizability of these findings and warrants cautious interpretation. Second, our analyses demonstrated substantial heterogeneity, primarily due to the inherent nature of the data [59, 60]. We used meta‐regression to identify the source of this heterogeneity. Therefore, caution should be exercised in interpreting the results. Future research should focus on conducting high‐quality studies to assess OME recurrence in children. Third, most of the studies included in our meta‐analysis were conducted predominantly in two continents, which could potentially limit the generalizability of OME recurrence results to other geographical regions. Therefore, conducting further research in various geographical regions is essential to confirm the global applicability of our findings. Fourth, the definition of recurrence was not standardized across the included studies. Specifically, criteria related to timing (e.g., minimum interval after tympanostomy tube extrusion) and severity (e.g., diagnostic confirmation methods or symptom thresholds) were not consistently reported or applied. As a result, recurrence was interpreted according to the definitions provided in the original studies, which may introduce heterogeneity and limit the comparability of findings.

## 5. Conclusion

The results of this systematic review and meta‐analysis demonstrated that the overall recurrence rate of OME following VT insertion in children is approximately 30%, with no significant difference observed between genders. Patients with cleft palate were found to have a higher likelihood of OME recurrence and therefore require long‐term follow‐up. Although some studies have suggested that the addition of antibiotics to VT insertion may reduce recurrence in children at risk for chronic infection, this evidence was not consistent across all included studies and should be interpreted with caution. Moreover, limitations such as small subgroup sample sizes, lack of individual patient data, potential publication bias, and the geographic concentration of available studies may restrict the generalizability of these findings. Future large‐scale, multicenter clinical trials are needed to confirm these observations and provide more definitive guidance for clinical practice.

## Author Contributions

A.R. and M.H.T.B.: conceptualization and study design. A.R., M.R.M., and M.M.: database search strategy and literature search. Z.T.B., M.H.T.B., and A.R.: study screening, selection, and quality assessment. A.R.: data analysis and interpretation. A.R., Z.T.B., and M.R.M.: manuscript drafting and editing. M.H.T.B. and M.R.M. contributed equally to this work and are jointly considered the first authors.

## Funding

No funding was received for this manuscript.

## Disclosure

All authors approved the submitted and final versions.

## Ethics Statement

Ethical approval was not required for this study in accordance with local/national guidelines. Written informed consent to participate in the study was not required in accordance with local/national guidelines. This study does not involve sensitive personal data, ethical issues, or policy breaches.

## Conflicts of Interest

The authors declare no conflicts of interest.

## Supporting Information

Additional supporting information can be found online in the Supporting Information section.

## Supporting information


**Supporting Information 1.** Appendix S1: Completed PRISMA 2020 checklist, which outlines adherence to the recommended reporting guidelines for systematic reviews and meta‐analyses.

**Supporting Information 2.** Appendix S2: Detailed search strategies used for each database, including the specific terms and Boolean operators applied in the literature search.

## Data Availability

The data that support the findings of this study are available on request from the corresponding author. The data are not publicly available due to privacy or ethical restrictions.

## References

[1] Rosenfeld R. M. , Shin J. J. , Schwartz S. R. , Coggins R. , Gagnon L. , Hackell J. M. , Hoelting D. , Hunter L. L. , Kummer A. W. , Payne S. C. , Poe D. S. , Veling M. , Vila P. M. , Walsh S. A. , and Corrigan M. D. , Clinical Practice Guideline: Otitis Media With Effusion (Update), Otolaryngology–Head and Neck Surgery. (2016) 154, S1–s41, 10.1177/0194599815623467. 26832942

[2] Walker R. E. , Bartley J. , Camargo C. A. , Flint D. , Thompson J. M. D. , and Mitchell E. A. , Higher Serum 25(OH)D Concentration Is Associated With Lower Risk of Chronic Otitis Media With Effusion: A Case-Control Study, Acta Paediatrica. (2017) 106, no. 9, 1487–1492, 10.1111/apa.13908, 28477429. 28477429

[3] Aarhus L. , Tambs K. , Kvestad E. , and Engdahl B. , Childhood Otitis Media: A Cohort Study With 30-Year Follow-Up of Hearing (The HUNT Study), Ear and hearing. (2015) 36, no. 3, 302–308, 10.1097/AUD.0000000000000118, 25401378. 25401378 PMC4409918

[4] Luotonen M. , Uhari M. , Aitola L. , Lukkaroinen A. M. , Luotonen J. , and Uhari M. , A Nation-Wide, Population-Based Survey of Otitis Media and School Achievement, International Journal of Pediatric Otorhinolaryngology. (1998) 43, no. 1, 41–51, 10.1016/S0165-5876(97)00157-2, 9596369. 9596369

[5] Berkman N. D. , Wallace I. F. , Steiner M. J. , Harrison M. , Greenblatt A. M. , Lohr K. N. , Kimple A. , and Yuen A. , Otitis Media With Effusion: Comparative Effectiveness of Treatments, AHRQ Comparative Effectiveness Reviews, 2013, Agency for Healthcare Research and Quality. 23762917

[6] Hosseini S. , Khajavi M. , Eftekharian A. , and Akbari N. , Vitamin D Levels in Children With Otitis Media With Effusion: A Case-Control Study, Thrita. (2016) 5, no. 1, e31977, 10.5812/thrita.31977.

[7] Lee J. Y. , Kim S. H. , Song C. I. , Kim Y. R. , Kim Y. J. , and Choi J. H. , Risk Factors for Persistent Otitis Media With Effusion in Children: A Case-Control Study, Yeungnam University Journal of Medicine. (2018) 35, no. 1, 70–75, 10.12701/yujm.2018.35.1.70, 31620573. 31620573 PMC6784671

[8] McCoy J. L. , Kaffenberger T. M. , Yang T. S. , and Dohar J. E. , Otitis Media Prone Children With Cystic Fibrosis: A New Normal, American Journal of Otolaryngology. (2021) 42, no. 5, 103137, 10.1016/j.amjoto.2021.103137, 34174638. 34174638 PMC8403146

[9] Akcan F. A. , Dündar Y. , Akcan H. B. , Uluat A. , Cebeci D. , Sungur M. A. , and Ünlü İ. , Clinical Role of Vitamin D in Prognosis of Otitis Media With Effusion, International Journal of Pediatric Otorhinolaryngology. (2018) 105, 1–5, 10.1016/j.ijporl.2017.11.030, 29447793. 29447793

[10] Núñez-Batalla F. , Jáudenes-Casaubón C. , Sequí-Canet J. M. , Vivanco-Allende A. , and Zubicaray-Ugarteche J. , Diagnosis and Treatment of Otitis Media With Effusion: CODEPEH Recommendations, Acta Otorrinolaringológica Española. (2019) 70, no. 1, 36–46, 10.1016/j.otorri.2017.07.004, 29033123. 29033123

[11] Charusripan P. and Khattiyawittayakun L. , The Effectiveness of Myringotomy and Ventilation Tube Insertion Versus Observation in Post-Radiation Otitis Media With Effusion, European Archives of Oto-Rhino-Laryngology. (2017) 274, no. 9, 3283–3290, 10.1007/s00405-017-4617-5, 28540514. 28540514

[12] Heidemann C. H. , Lauridsen H. H. , Kjeldsen A. D. , Faber C. E. , Johansen E. C. , and Godballe C. , Quality-of-Life Differences Among Diagnostic Subgroups of Children Receiving Ventilating Tubes for Otitis Media, Otolaryngology--Head and Neck Surgery. (2015) 153, no. 4, 636–643, 10.1177/0194599815569491, 25676152. 25676152

[13] Takai S. , Nomura K. A.-O. , Oda K. , Ozawa D. , Irimada M. , Ikeda R. A.-O. , Kakuta R. , Katori Y. , and Ohyama K. , Clinical Factors Associated With the Outcomes of Long-Term Middle Ear Ventilation Tube Insertion in Pediatric Patients, Ear Nose & Throat Journal. (2023) 102, no. 10, NP511–NP517, 10.1177/01455613211026437, 34130509. 34130509

[14] MacKeith S. , Mulvaney C. A. , Galbraith K. , Webster K. E. , Connolly R. , Paing A. , Marom T. , Daniel M. , Venekamp R. P. , Rovers M. M. , and Schilder A. G. , Ventilation Tubes (Grommets) for Otitis Media With Effusion (OME) in Children, Cochrane Database of Systematic Reviews. (2023) 11, no. 11, CD015215, 10.1002/14651858.CD015215.pub2, 37965944. 37965944 PMC10646987

[15] Alaraifi A. K. , Alkhaldi A. S. , Ababtain I. S. , and Alsaab F. , Predictors of Otitis Media With Effusion Recurrence Following Myringotomy, Indian Journal of Otolaryngology and Head and Neck Surgery. (2022) 74, no. supplement 3, 4053–4058, 10.1007/s12070-021-02817-0, 36742680. 36742680 PMC9895307

[16] Klopp-Dutote N. , Kolski C. , Strunski V. , and Page C. , Tympanostomy Tubes for Serous Otitis Media and Risk of Recurrences, International Journal of Pediatric Otorhinolaryngology. (2018) 106, 105–109, 10.1016/j.ijporl.2018.01.023. 29447881

[17] Mandel E. M. , Swarts J. D. , Casselbrant M. L. , Tekely K. K. , Richert B. C. , Seroky J. T. , and Doyle W. J. , Eustachian Tube Function as a Predictor of the Recurrence of Middle Ear Effusion in Children, Laryngoscope. (2013) 123, no. 9, 2285–2290, 10.1002/lary.24021, 23575552. 23575552 PMC3711968

[18] Hao J. , Chen M. , Liu B. , Yang Y. , Liu W. , Ma N. , Han Y. , Liu Q. , Ni X. , and Zhang J. , Compare Two Surgical Interventions for Otitis Media With Effusion in Young Children, European Archives of Oto-rhino-laryngology. (2019) 276, no. 8, 2125–2131, 10.1007/s00405-019-05421-9, 31127413. 31127413

[19] Songu M. , Islek A. , Imre A. , Aslan H. , Aladag I. , Pinar E. , and Oncel S. , Risk Factors for Otitis Media With Effusion in Children With Adenoid Hypertrophy, Acta Otorhinolaryngologica Italica. (2020) 40, no. 2, 133–137, 10.14639/0392-100X-2456, 32469007. 32469007 PMC7256910

[20] Lodge C. J. , Bowatte G. , Matheson M. C. , and Dharmage S. C. , The Role of Breastfeeding in Childhood Otitis Media, Current Allergy and Asthma Reports. (2016) 16, no. 9, 10.1007/s11882-016-0647-0, 27595154. 27595154

[21] Ponduri S. , Bradley R. , Ellis P. E. , Brookes S. T. , Sandy J. R. , and Ness A. R. , The Management of Otitis Media With Early Routine Insertion of Grommets in Children With Cleft Palate -- A Systematic Review, Cleft Palate-Craniofacial Journal. (2009) 46, no. 1, 30–38, 10.1597/07-219.1, 19115800. 19115800

[22] Flynn T. , Möller C. , Jönsson R. , and Lohmander A. , The High Prevalence of Otitis Media With Effusion in Children With Cleft Lip and Palate as Compared to Children Without Clefts, International Journal of Pediatric Otorhinolaryngology. (2009) 73, no. 10, 1441–1446, 10.1016/j.ijporl.2009.07.015, 19709760. 19709760

[23] Yoo M. H. , Cho Y. S. , Choi J. , Choung Y. H. , Chung J. H. , Chung J. W. , Han G. C. , Jun B. C. , Kim D. K. , Kim K. S. , Lee J. H. , Lee K. Y. , Lee S. H. , Moon I. S. , Park H. J. , Park S. N. , Rhee J. , Seo J. H. , and Yeo S. G. , Factors Affecting the Extrusion Rate and Complications After Ventilation Tube Insertion: A Multicenter Registry Study on the Effectiveness of Ventilation Tube Insertion in Pediatric Patients With Chronic Otitis Media With Effusion-Part II, Clinical and Experimental Otorhinolaryngology. (2022) 15, no. 4, 326–334, 10.21053/ceo.2022.00934, 36097840. 36097840 PMC9723292

[24] Munn Z. , Moola S. , Lisy K. , Riitano D. , and Tufanaru C. , Methodological Guidance for Systematic Reviews of Observational Epidemiological Studies Reporting Prevalence and Cumulative Incidence Data, JBI Evidence Implementation. (2015) 13, no. 3, 147–153, 10.1097/XEB.0000000000000054, 26317388. 26317388

[25] Saad K. , Abdelmoghny A. , Abdel-Raheem Y. F. , Gad E. F. , and Elhoufey A. , Prevalence and Associated Risk Factors of Recurrent Otitis Media With Effusion in Children in Upper Egypt, World Journal of Otorhinolaryngology-Head and Neck Surgery. (2021) 7, no. 4, 280–284, 10.1016/j.wjorl.2020.08.002, 34632340. 34632340 PMC8486686

[26] Yaman H. , Yilmaz S. , Guclu E. , Subasi B. , Alkan N. , and Ozturk O. , Otitis Media With Effusion: Recurrence After Tympanostomy Tube Extrusion, International Journal of Pediatric Otorhinolaryngology. (2010) 74, no. 3, 271–274, 10.1016/j.ijporl.2009.11.035. 20044147

[27] De Felice C. , De Capua B. , Costantini D. , Martufi C. , Toti P. , Tonni G. , Laurini R. , Giannuzzi A. , and Latini G. , Recurrent Otitis Media With Effusion in Preterm Infants With Histologic Chorioamnionitis - A 3 Years Follow-Up Study, Early Human Development. (2008) 84, no. 10, 667–671, 10.1016/j.earlhumdev.2008.04.008, 18760552. 18760552

[28] Engel J. A. M. , Straetemans M. , and Zielhuis G. A. , Birth Characteristics and Recurrent Otitis Media With Effusion in Young Children, International Journal of Pediatric Otorhinolaryngology. (2005) 69, no. 4, 533–540, 10.1016/j.ijporl.2004.11.026, 15763293. 15763293

[29] Xenellis J. , Paschalidis J. , Georgalas C. , Davilis D. , Tzagaroulakis A. , and Ferekidis E. , Factors Influencing the Presence of Otitis Media With Effusion 16 Months After Initial Diagnosis in a Cohort of School-Age Children in Rural Greece: A Prospective Study, International Journal of Pediatric Otorhinolaryngology. (2005) 69, no. 12, 1641–1647, 10.1016/j.ijporl.2005.03.047, 15941593. 15941593

[30] Ovesen T. , Felding J. U. , Tommerup B. , Schousboe L. P. , and Petersen C. G. , Effect of N-Acetylcysteine on the Incidence of Recurrence of Otitis Media With Effusion and Re-Insertion of Ventilation Tubes, Acta Oto-Laryngologica Supplementum. (2000) 543, no. 543, 79–81, 10.1080/000164800454044, 10908985. 10908985

[31] Popova D. , Varbanova S. , and Popov T. M. , Comparison Between Myringotomy and Tympanostomy Tubes in Combination With Adenoidectomy in 3-7-Year-Old Children With Otitis Media With Effusion, International Journal of Pediatric Otorhinolaryngology. (2010) 74, no. 7, 777–780, 10.1016/j.ijporl.2010.03.054, 20399511. 20399511

[32] Baljošević I. , Čvorović L. , Stanković K. , Šubarević V. , and Baljošević Z. , Risk Factors for Recurrent Otitis Media With Effusion, Vojnosanitetski Pregled. (2017) 74, no. 12, 1117–1120, 10.2298/VSP151201308B.

[33] Rieu-Chevreau C. , Lavagen N. , Gbaguidi C. , Dakpé S. , Klopp-Dutote N. , and Page C. , Risk of Occurrence and Recurrence of Otitis Media With Effusion in Children Suffering From Cleft Palate, International journal of pediatric otorhinolaryngology.(2019) 120, 1–5, 10.1016/j.ijporl.2019.01.041, 30735917. 30735917

[34] Iemura-Kashiwagi M. , Okano T. , Iwai N. , Taniguchi M. , and Omori K. , Prognosis of Otitis Media With Effusion in Pediatric Patients With Cleft Palate During Language-Acquisition Period Treated by Simultaneous Tympanostomy Tube Placement With Palatoplasty, International Journal of Pediatric Otorhinolaryngology. (2022) 155, 111071, 10.1016/j.ijporl.2022.111071, 35217270. 35217270

[35] Luo H.-N. , Ma S.-J. , Sheng Y. , Yan J. , Hou J. , Zhu K. , and Ren X.-Y. , Pepsin Deteriorates Prognosis of Children With Otitis Media With Effusion Who Undergo Myringotomy or Tympanostomy Tube Insertion, International Journal of Pediatric Otorhinolaryngology. (2014) 78, no. 12, 2250–2254, 10.1016/j.ijporl.2014.10.026. 25465449

[36] Suwansa-Ard S. , Chortrakarnkij P. , and Vathanophas V. , Long Term Study of Otitis Media With Effusion After Palatoplasty and Myringotomy With Ventilation Tube Insertion, Journal of Health Science and Medical Research. (2020) 41, no. 3, 2023924, 10.31584/jhsmr.2023924.

[37] Shareef A. , Langenfeld T. , Hill M. , Vachhrajani S. , and Elluru R. , Efficacy of Tympanostomy Tube Placement With Adjuvant Adenoidectomy in Children Less Than 4 Years of Age, International Journal of Pediatric Otorhinolaryngology. (2024) 176, 111823, 10.1016/j.ijporl.2023.111823, 38134590. 38134590

[38] Ha J. , Gu G. Y. , Yeou S. H. , Kim H. , Choo O.-S. , Jang J. H. , Park H. Y. , and Choung Y. H. , Determination of Tympanostomy Tube Types for Otitis Media With Effusion in Patients With Cleft Palate: Comparison Between Paparella Type 1 and Type 2 Tubes, Journal of Clinical Medicine. (2023) 12, no. 20, 10.3390/jcm12206651, 37892790. PMC10607012 37892790

[39] Jia D. , Chen Y. , Wang X. , Xu G. , Chen J. , Li L. , Pan H. , and Wu Z. , Outcomes and Prognostic Factors of Balloon Eustachian Tuboplasty Combined With Ventilation Tubes Insertion in Children: A Retrospective Study, Ear Nose and Throat Journal. (2026) 105, no. 3, NP240–NP250, 10.1177/01455613231188295, 37515366. 37515366

[40] Alaraifi A. K. , Alosfoor M. A. , and Alsaab F. , Impact of Pediatric Obesity on the Prevalence and Outcome of Otitis Media With Effusion, International Journal of Pediatric Otorhinolaryngology. (2020) 133, 110005, 10.1016/j.ijporl.2020.110005, 32213420. 32213420

[41] Straetemans M. , van Heerbeek N. , Sanders E. A. , Engel J. A. , Schilder A. G. , Rijkers G. T. , Graamans K. , Straatman H. , and Zielhuis G. A. , Immune Status and Eustachian Tube Function in Recurrence of Otitis Media With Effusion, Archives of Otolaryngology--Head and Neck Surgery. (2005) 131, no. 9, 771–776, 10.1001/archotol.131.9.771, 16172352. 16172352

[42] Taziki et al., Recurrence and Associated Factors in Otitis Media With Effusion (OME) Patients After Surgery in Northeast Iran: A Retrospective Cohort Study, World Journal of Otorhinolaryngology - Head and Neck Surgery. Under Review. (2025) R1.

[43] Mandel E. M. , Rockette H. E. , Bluestone C. D. , Paradise J. L. , and Nozza R. J. , Efficacy of Myringotomy With and Without Tympanostomy Tubes for Chronic Otitis Media With Effusion, Pediatric Infectious Disease Journal. (1992) 11, no. 4, 270–277, 10.1097/00006454-199204000-00003, 1565550. 1565550

[44] Boston M. , McCook J. , Burke B. , and Derkay C. , Incidence of and Risk Factors for Additional Tympanostomy Tube Insertion in Children, Archives of Otolaryngology--Head and Neck Surgery. (2003) 129, no. 3, 293–296, 10.1001/archotol.129.3.293, 12622537. 12622537

[45] Martines F. , Bentivegna D. , Maira E. , Sciacca V. , and Martines E. , Risk Factors for Otitis Media With Effusion: Case-Control Study in Sicilian Schoolchildren, International Journal of Pediatric Otorhinolaryngology. (2011) 75, no. 6, 754–759, 10.1016/j.ijporl.2011.01.031, 21514964. 21514964

[46] Rosenfeld R. M. , Tunkel D. E. , Schwartz S. R. , Anne S. , Bishop C. E. , Chelius D. C. , Hackell J. , Hunter L. L. , Keppel K. L. , Kim A. H. , Kim T. W. , Levine J. M. , Maksimoski M. T. , Moore D. J. , Preciado D. A. , Raol N. P. , Vaughan W. K. , Walker E. A. , and Monjur T. M. , Executive Summary of Clinical Practice Guideline on Tympanostomy Tubes in Children (Update), Otolaryngology–Head and Neck Surgery. (2022) 166, no. 2, 189–206, 10.1177/01945998211065661, 35138976. 35138976

[47] Rovers M. M. , Zielhuis G. A. , Straatman H. , Ingels K. , van der Wilt G. J. , and van den Broek P. , Prognostic Factors for Persistent Otitis Media With Effusion in Infants, Archives of Otolaryngology--Head and Neck Surgery. (1999) 125, no. 11, 1203–1207, 10.1001/archotol.125.11.1203. 10555690

[48] Assimon M. M. , Confounding in Observational Studies Evaluating the Safety and Effectiveness of Medical Treatments, Kidney360. (2021) 2, no. 7, 1156–1159, 10.34067/KID.0007022020, 35368357. 35368357 PMC8786092

[49] Sedgwick P. , Randomised Controlled Trials: Understanding Confounding, BMJ. (2015) 351, 1756–1833, 10.1136/bmj.h5119. 26408098

[50] Pichichero M. E. , Recurrent and Persistent Otitis Media, Pediatric Infectious Disease Journal. (2000) 19, no. 9, 911–916, 10.1097/00006454-200009000-00034. 11001126

[51] Daniel M. , Imtiaz-Umer S. , Fergie N. , Birchall J. P. , and Bayston R. , Bacterial Involvement in Otitis Media With Effusion, International Journal of Pediatric Otorhinolaryngology. (2012) 76, no. 10, 1416–1422, 10.1016/j.ijporl.2012.06.013. 22819485

[52] Coates H. , Thornton R. , Langlands J. , Filion P. , Anthony D. K. , Vijayasekaran S. , and Richmond P. , The Role of Chronic Infection in Children With Otitis Media With Effusion: Evidence for Intracellular Persistence of Bacteria, Otolaryngology—Head and Neck Surgery. (2008) 138, no. 6, 778–781, 10.1016/j.otohns.2007.02.009, 18503854. 18503854

[53] Gates G. A. , Avery C. A. , Prihoda T. J. , and Cooper J. C. , Effectiveness of Adenoidectomy and Tympanostomy Tubes in the Treatment of Chronic Otitis Media With Effusion, New England Journal of Medicine. (1987) 317, no. 23, 1444–1451, 10.1056/NEJM198712033172305. 3683478

[54] Paradise J. L. , Bluestone C. D. , Rogers K. D. , Taylor F. H. , Colborn D. K. , Bachman R. Z. et al., Efficacy of Adenoidectomy for Recurrent Otitis Media in Children Previously Treated With Tympanostomy-Tube Placement Results of Parallel Randomized and Nonrandomized Trials, Jama. (1990) 263, no. 15, 2066–2073, 10.1001/jama.1990.03440150074029, 2181158. 2181158

[55] Paradise J. L. , Bluestone C. D. , Colborn D. K. , Bernard B. S. , Smith C. G. , Rockette H. E. , and Kurs-Lasky M. , Adenoidectomy and Adenotonsillectomy for Recurrent Acute Otitis Media: Parallel Randomized Clinical Trials in Children Not Previously Treated With Tympanostomy Tubes, Jama. (1999) 282, no. 10, 945–953, 10.1001/jama.282.10.945, 10485679. 10485679

[56] Bluestone C. D. and Klein J. O. , Otitis Media in Infants and Children, 2007, PMPH-USA.

[57] Yoshitomi A. A.-O. , Baba S. , Tamada I. , Nakaya M. , and Itokawa M. , Relationship Between Cleft Palate Width and Otitis Media, Laryngoscope Investigative Otolaryngology. (2022) 7, no. 6, 2126–2132, 10.1002/lio2.933, 36544954. 36544954 PMC9764805

[58] Jeong J. , Lim H. , Eo T. S. , Lee K. , Oh J. , and Choi H. S. , Effects of Adenoidectomy and Adenotonsillectomy on Tympanostomy Tube Reinsertion Based on Korean Population-Based National Sample Cohort Data, Journal of International Advanced Otology. (2020) 16, no. 3, 387–392, 10.5152/iao.2020.8862, 33136022. 33136022 PMC7901457

[59] Barker T. A.-O. X. , Migliavaca C. B. , Stein C. , Colpani V. , Falavigna M. , Aromataris E. , and Munn Z. , Conducting Proportional Meta-Analysis in Different Types of Systematic Reviews: A Guide for Synthesisers of Evidence, BMC Medical Research Methodology. (2021) 21, no. 1, 10.1186/s12874-021-01381-z, 34544368. PMC8451728 34544368

[60] von Hippel P. T. , The Heterogeneity Statistic I(2) Can Be Biased in Small Meta-Analyses, BMC Medical Research Methodology. (2015) 15, no. 1, 10.1186/s12874-015-0024-z, 25880989. PMC4410499 25880989

