# Supplementary material for: Incidence of Recurrent Otitis Media With Effusion (OME) Following Tympanostomy in Pediatric Patients: A Systematic Review and Cumulative Meta‐Analysis
Source: Int J Pediatr. 2026 Jun 24;2026:8886046. doi: 10.1155/ijpe/8886046 (PMC13292024; doi:10.1155/ijpe/8886046)
Supplement: Supplementary file 2 — Supporting Information 2. Appendix S2: Detailed search strategies used for each database, including the specific terms and Boolean operators applied in the literature search. [file IJPE-2026-8886046-s002.docx]

August 2024

Main Syntax

**(Incidence* OR "Attack Rate*" OR "Person time Rate*" OR Prevalence* OR Risk*) AND ("Serous Otitis Media" OR "Secretory Otitis Media" OR "Middle Ear Effusion*" OR "Otitis Media with Effusion") AND (Recurrence* OR Relapse*)**

| Database | Formula | Results |
| --- | --- | --- |
| PubMed | (((Incidence*[Title/Abstract] OR "Attack Rate*"[Title/Abstract] OR "Person time Rate*"[Title/Abstract] OR Prevalence*[Title/Abstract] OR Risk*[Title/Abstract]) AND ("Serous Otitis Media"[Title/Abstract] OR "Secretory Otitis Media"[Title/Abstract] OR "Middle Ear Effusion*"[Title/Abstract] OR "Otitis Media with Effusion"[Title/Abstract])) AND (Recurrence*[Title/Abstract] OR Relapse*[Title/Abstract])) OR (((("Incidence"[Mesh]) OR "Prevalence"[Mesh]) OR "Risk"[Mesh]) AND (("Recurrence"[Mesh]) AND "Otitis Media with Effusion"[Mesh])) | 182 |
| Web of Science | (Incidence* OR "Attack Rate*" OR "Person time Rate*" OR Prevalence* OR Risk*) AND ("Serous Otitis Media" OR "Secretory Otitis Media" OR "Middle Ear Effusion*" OR "Otitis Media with Effusion") AND (Recurrence* OR Relapse*) (Topic)  Timespan: All years. Indexes: SCI-EXPANDED, SSCI, A&HCI, CPCI-S, CPCI-SSH, BKCI-S, BKCI-SSH, ESCI. | 90 |
| Scopus | TITLE-ABS-KEY ( ( incidence* OR "Attack Rate*" OR "Person time Rate*" OR prevalence* OR risk* ) AND ( "Serous Otitis Media" OR "Secretory Otitis Media" OR "Middle Ear Effusion*" OR "Otitis Media with Effusion" ) AND ( recurrence* OR relapse* ) ) | 366 |
| Embase | ((incidence*:ti,ab,kw OR 'attack rate*':ti,ab,kw OR 'person time rate*':ti,ab,kw OR prevalence*:ti,ab,kw OR risk*:ti,ab,kw) AND ('serous otitis media':ti,ab,kw OR 'secretory otitis media':ti,ab,kw OR 'middle ear effusion*':ti,ab,kw OR 'otitis media with effusion':ti,ab,kw) AND (recurrence*:ti,ab,kw OR relapse*:ti,ab,kw)) OR (('incidence'/exp OR 'prevalence'/exp OR 'risk'/exp OR 'attack rate'/exp OR 'incidence density rate'/exp) AND ('serous otitis media'/exp) AND ('recurrent disease'/exp OR 'relapse'/exp)) | 118 |
| Cochrane | (((Incidence* OR "Attack Rate*" OR "Person time Rate*" OR Prevalence* OR Risk*) AND ("Serous Otitis Media" OR "Secretory Otitis Media" OR "Middle Ear Effusion*" OR "Otitis Media with Effusion") AND (Recurrence* OR Relapse*)):ti,ab,kw) OR ((MeSH descriptor: [Incidence] explode all trees OR MeSH descriptor: [Prevalence] explode all trees OR MeSH descriptor: [Risk] explode all trees) AND MeSH descriptor: [Otitis Media with Effusion] explode all trees AND MeSH descriptor: [Recurrence] explode all trees) | 50 (47 trials, 3 review) |
| ProQuest | abstract((Incidence* OR "Attack Rate*" OR "Person time Rate*" OR Prevalence* OR Risk*) AND ("Serous Otitis Media" OR "Secretory Otitis Media" OR "Middle Ear Effusion*" OR "Otitis Media with Effusion") AND (Recurrence* OR Relapse*)) OR subject((Incidence* OR "Attack Rate*" OR "Person time Rate*" OR Prevalence* OR Risk*) AND ("Serous Otitis Media" OR "Secretory Otitis Media" OR "Middle Ear Effusion*" OR "Otitis Media with Effusion") AND (Recurrence* OR Relapse*)) OR title((Incidence* OR "Attack Rate*" OR "Person time Rate*" OR Prevalence* OR Risk*) AND ("Serous Otitis Media" OR "Secretory Otitis Media" OR "Middle Ear Effusion*" OR "Otitis Media with Effusion") AND (Recurrence* OR Relapse*)) | 202 |
| Wiley | ("(Incidence* OR "Attack Rate*" OR "Person time Rate*" OR Prevalence* OR Risk*) AND ("Serous Otitis Media" OR "Secretory Otitis Media" OR "Middle Ear Effusion*" OR "Otitis Media with Effusion") AND (Recurrence* OR Relapse*)" in Keywords) OR ("(Incidence* OR "Attack Rate*" OR "Person time Rate*" OR Prevalence* OR Risk*) AND ("Serous Otitis Media" OR "Secretory Otitis Media" OR "Middle Ear Effusion*" OR "Otitis Media with Effusion") AND (Recurrence* OR Relapse*)" in Abstract) OR ("(Incidence* OR "Attack Rate*" OR "Person time Rate*" OR Prevalence* OR Risk*) AND ("Serous Otitis Media" OR "Secretory Otitis Media" OR "Middle Ear Effusion*" OR "Otitis Media with Effusion") AND (Recurrence* OR Relapse*)" in Title) | 8 |
| Ovid | ((Incidence* or "Attack Rate*" or "Person time Rate*" or Prevalence* or Risk*) and ("Serous Otitis Media" or "Secretory Otitis Media" or "Middle Ear Effusion*" or "Otitis Media with Effusion") and (Recurrence* or Relapse*)).at. or ((Incidence* or "Attack Rate*" or "Person time Rate*" or Prevalence* or Risk*) and ("Serous Otitis Media" or "Secretory Otitis Media" or "Middle Ear Effusion*" or "Otitis Media with Effusion") and (Recurrence* or Relapse*)).ab. or ((Incidence* or "Attack Rate*" or "Person time Rate*" or Prevalence* or Risk*) and ("Serous Otitis Media" or "Secretory Otitis Media" or "Middle Ear Effusion*" or "Otitis Media with Effusion") and (Recurrence* or Relapse*)).kw. | 101 |
| Total= 1117  After deleting duplicated files= 623 | |  |
